# La reforma en salud del Ecuador

**DOI:** 10.26633/RPSP.2017.96

**Published:** 2017-05-15

**Authors:** Verónica Espinosa, Cecilia Acuña, Daniel de la Torre, Gina Tambini

**Affiliations:** 1 Ministerio de Salud Pública de Ecuador >Ministerio de Salud Pública de Ecuador Ministerio de Salud Pública de Ecuador, Quito, Ecuador.; 2 Organización Panamericana de la Salud/Organización Mundial de la Salud Organización Panamericana de la Salud/Organización Mundial de la Salud Organización Panamericana de la Salud/Organización Mundial de la Salud, Quito, Ecuador.

Para comprender el proceso de reforma del sector salud en el Ecuador, es necesario partir del marco normativo e ideológico sobre el cual se basa la transformación sanitaria de la última década.

A partir del año 2008 Ecuador reconoce en su Constitución el derecho a la salud con una visión sistémica, vinculándolo con el ejercicio de otros derechos. La Carta Magna del 2008 establece al Estado como garante del derecho a la salud a través de la formulación de políticas, planes y programas orientados a brindar acceso a servicios de promoción y atención integral bajo los principios de equidad, universalidad, solidaridad, interculturalidad, calidad, eficiencia, eficacia, precaución y bioética, con enfoque de género y generacional (1). De este modo, el mandato constitucional determina la necesidad de iniciar el proceso de reforma y define el ámbito en el cual se conduciría dicho proceso para lograr que el Estado cumpla con las nuevas obligaciones establecidas en la Constitución.

Ello requirió en primer lugar de la construcción de un nuevo modelo de atención ya no centrado en la enfermedad o en la prestación de servicios curativos, sino en las personas, sus familias, sus comunidades y sus necesidades de salud, con una perspectiva de promoción, prevención, recuperación y rehabilitación. El Modelo de Atención Integral en Salud (MAIS) (2) se constituyó en un eje orientador de la reforma del sector y en un pilar de la reorganización institucional del sistema público de salud, aun cuando su implementación en el nivel local ha resultado más compleja de lo esperado y a la fecha no se ha completado.

Fiel al mandato constitucional, el Ministerio de Salud Pública (MSP) instauró la gratuidad de los servicios de salud para todos los usuarios de su red de prestación. El enorme incremento de la demanda por servicios generado por la gratuidad y la situación de abandono en la que se encontraba la infraestructura pública de salud desde hacía más de 40 años, planteó la urgente necesidad de modificar los criterios de despliegue territorial de los establecimientos de salud y de mejorar la infraestructura sanitaria disponible. Con este fin se implementó una metodología de planificación territorial basada en la cantidad de población existente en cada unidad geográfica y en la distancia ideal a la cual deberían situarse los centros de salud para permitir un acceso adecuado y expedito. Se definieron tipologías de establecimientos con una capacidad de resolución y una cartera de servicios diferenciada para el primer, segundo y tercer nivel de atención; se construyeron estándares para el licenciamiento de dichos establecimientos; se revisaron y adecuaron los modelos de gestión, los manuales de puestos y los diseños para la construcción y adecentamiento de obras; se adquirió equipamiento y se contrataron recursos humanos de acuerdo a la cantidad de nuevos establecimientos definida por la planificación territorial. Con todo este arsenal de nuevos instrumentos se construyeron, repararon y pusieron en marcha 47 hospitales y 74 centros de salud de primer nivel, permitiendo absorber un incremento de más de 300% en la demanda por servicios de salud, con una inversión total de US$ 16 208 millones entre el 2007 y el 2016 (3) y una inversión promedio anual cinco veces superior al periodo 2000–2006 (4).

Un elemento fundamental del proceso de reforma fue la recuperación de la rectoría del MSP sobre el sector salud. Ello se logró mediante profundos cambios institucionales en dos ámbitos: en la estructura del Ministerio, con el desarrollo del Estatuto Orgánico por procesos (5) y en la arquitectura de relacionamiento entre las instituciones prestadoras de servicios de salud. La definición de una nueva estructura orgánica del MSP con dos viceministerios, nueve coordinaciones zonales y 140 distritos distribuidos en todo el territorio nacional, fortaleció su presencia a nivel nacional y local. La separación explícita del rol rector y el rol prestador del MSP, radicados cada uno en un viceministerio (el primero en el Viceministerio de Gobernanza de la Salud Pública y el segundo en el Viceministerio de Atención Integralde Salud) permitió visibilizar y dimensionar por primera vez la rectoría como función esencial de un Ministerio que históricamente se percibía a sí mismo como prestador de servicios y no como la autoridad sanitaria nacional.

La creación de dos agencias reguladoras, una para los productos de uso y consumo humano (ARCSA) y la otra para la calidad de los prestadores y aseguradores de salud (ACESS), así como la extensa normativa sanitaria emitida para todo el sistema de salud por el Viceministerio de Gobernanza –tales como guías de práctica clínica, reglamentos e instructivos–, permitió la consolidación del marco regulatorio en un sector previamente caracterizado por la desregulación. En este ámbito destaca el Reglamento Sanitario del Etiquetado de Alimentos Procesados para el Consumo Humano (“semáforo de alimentos”) (6), cuya promulgación convirtió a Ecuador en país líder en la emisión de este tipo de normativa en la Región de las Américas, constituyéndose en uno de los ejes del posicionamiento del país en la agenda de salud global en el periodo 2011–2015.

Por su parte, la creación de la Red Pública Integral de Salud y sus mecanismos de relacionamiento, establecidos en el convenio marco entre las diversas instituciones prestadoras del subsistema público (7), así como el Tarifario Nacional de Prestaciones, que rige la compra de servicios entre los prestadores públicos y entre el sistema público y el privado, han permitido dar pasos concretos en la reducción de la segmentación y la fragmentación del sistema de salud y avanzar hacia una integración cada vez mayor entre los subsistemas. Estos avances se consolidarán de forma definitiva una vez aprobado el nuevo Código Orgánico de la Salud, el cual constituye el marco legal que cristaliza el proceso de reforma en el ámbito legislativo.

Otro aspecto fundamental de la reforma es la gran inversión realizada en recursos humanos y el desarrollo de diversas estrategias destinadas a cubrir la brecha de profesionales de la salud, entre las cuales se encuentran el programa “Ecuador saludable por ti vuelvo”, orientado a promover el retorno de profesionales de la salud ecuatorianos para cubrir el déficit a corto plazo; la creación de la carrera de Técnico de Atención Primaria de Salud y el financiamiento de más de 1 500 becas para su formación; el reconocimiento del médico familiar como especialista, con un incremento acorde de su salario; la entrega de más de 3 600 becas de especialización; la creación del puesto de gerente de hospital para los hospitales de más de 70 camas; y la contratación de más de 5 000 profesionales de la salud entre 2012 y 2015. Como resultado de la implementación de estas estrategias, en el periodo 2008–2015 se triplicó el número de profesionales de la salud contratado por el MSP, pasando de 11 201 a 33 644, y se cuadruplicó su salario, el cual se ubicaba en un rango de US$ 919 a US$ 1 197 mensuales y se elevó a un rango de US$ 1 676 a US$ 4 000 mensuales con bono de residencia (4).

El sistema de salud ecuatoriano sin duda se ha fortalecido con el profundo y ambicioso proceso de reforma, el cual ha sido posible gracias a un apoyo político explícito y sostenido, expresado en la priorización de la salud como un derecho de todos y de todas. No obstante, existen grandes retos aún pendientes, entre los cuales se destacan la implementación de un modelo de financiamiento sostenible con un fondo mancomunado para el sistema público, que permita una mayor eficiencia en el gasto en salud y garantice la sostenibilidad del sistema en el mediano plazo; el fortalecimiento de la vigilancia epidemiológica y del sistema de información en salud, a fin de detectar de manera temprana y oportuna los brotes epidémicos y las enfermedades crónicas no transmisibles evitando que éstas se transformen en enfermedades catastróficas; la consolidación de las estrategias de prevención y control –las cuales requieren fortalecerse en el marco del proceso de reforma–; la implementación del MAIS y su materialización en rutinas de atención para los enfermos agudos pero sobre todo para los portadores de enfermedades crónicas no transmisibles, a fin de asegurar la continuidad de los cuidados; y la construcción efectiva de redes integradas de provisión de servicios de salud con mecanismos estables y eficientes de referencia y contrarreferencia.

Estas son las tareas a abordar en el futuro inmediato como parte de la consolidación de la reforma sanitaria, y como parte de la ruta del sistema de salud ecuatoriano hacia la salud universal.
